# APN Inhibitor Bestatin Induces MM Cell Differentiation Through the CD79B/BTK/STAT3 Pathway

**DOI:** 10.3390/cells15100949

**Published:** 2026-05-21

**Authors:** Xiaoke Wang, Chunyan Fang, Shanyu Li, Huakai Zeng, Junyi Liu, Xinwei Duan, Xiaoyi Zhang, Wenyan Jiang, Xuejian Wang

**Affiliations:** School of Pharmacy, Shandong Second Medical University, Weifang 261000, China; wangxiaoke959995@163.com (X.W.); 15155824252@163.com (S.L.); 18559486143@163.com (H.Z.); liu9981111@163.com (J.L.); 15552151536@163.com (X.D.); paisley14@163.com (X.Z.); jwy5858@163.com (W.J.)

**Keywords:** multiple myeloma, differentiation, Bestatin, CD79B/BTK

## Abstract

**Highlights:**

- This study is the first to report that the APN inhibitor Bestatin induces multiple myeloma (MM) cell differentiation via the CD79B/BTK-STAT3 axis, a novel regulatory mechanism filling the gap in understanding APN inhibitors’ role in MM differentiation.
- This study first demonstrates that Bestatin, ibrutinib (CD79B/BTK inhibitor) or GCDA (STAT3 agonist) synergizes with the clinical drug Ixazomib against MM, providing a novel combination strategy that may enhance therapeutic efficacy with theoretical and translational value.

**Abstract:**

Differentiation therapy holds significant potential for the treatment of multiple myeloma (MM). We previously identified that the aminopeptidase N (APN) inhibitor Bestatin promotes MM cell differentiation. Herein, we elucidate the underlying molecular mechanisms of this process. Utilizing MM1.S, U266, and RPMI-8226 cell lines, a combination of CCK-8 assays, flow cytometry, Wright–Giemsa staining, Western blotting, qRT-PCR, ELISA, APN enzymatic activity analysis, SA-β-gal staining, and bioinformatic analyses revealed elevated APN expression across all cell types. Bestatin treatment induced MM cell differentiation in a concentration-dependent manner, which was accompanied by the upregulation of the differentiation marker CD49e, increased immunoglobulin light chain secretion, elevated cellular senescence, and a concomitant suppression of cell proliferation and APN enzymatic activity. Mechanistically, Bestatin exerts its effects by downregulating the CD79B/BTK signaling pathway, thereby activating the downstream transcription factor STAT3. Consistent with this axis, direct inhibition of CD79B/BTK alone was sufficient to induce differentiation, while blockade of STAT3 completely abrogated the differentiation-promoting effect of Bestatin. The APN-neutralizing antibody (WM15) yielded consistent results with Bestatin, further validating this regulatory axis. Furthermore, both the CD79B/BTK inhibitor Ibrutinib and the STAT3 agonist GCDA potentiated the cytotoxicity of the clinical MM drug Ixazomib. Bestatin itself synergized with Ixazomib and enhanced the anti-proliferative effect of IL-6. In summary, our findings establish that the APN inhibitor Bestatin induces MM cell differentiation via the CD79B/BTK-STAT3 signaling axis. Targeting this pathway represents a promising strategy to enhance the efficacy of Ixazomib, providing a compelling rationale for novel combination therapies in MM.

## 1. Introduction

Multiple myeloma (MM) is a prevalent hematologic malignancy characterized by the clonal expansion of plasma cells within the bone marrow [1,2,3]. This pathological process leads to a range of clinical symptoms, including osteolytic bone destruction and impaired immune function [4,5]. Despite marked improvements in patient survival with the introduction of proteasome inhibitors and immunomodulatory drugs in recent years [6,7,8,9], relapse, and the emergence of drug resistance continue to pose formidable clinical challenges. Consequently, there is a pressing need to identify novel therapeutic targets and develop more effective treatment strategies.

Induction differentiation therapy represents a promising approach that redirects neoplastic cells toward a mature, less proliferative state. This strategy exerts its anti-tumor effects by suppressing proliferative capacity and diminishing invasive potential [10,11,12]. The underlying principle centers on compelling tumor cells to undergo terminal differentiation, thereby arresting their malignant progression [11]. A landmark achievement is its remarkable success in achieving durable remissions in acute promyelocytic leukemia [13,14]. A pivotal unresolved question is whether this differentiation-inducing strategy can be translated into effective treatment for MM.

Aminopeptidase N (APN) is a zinc-dependent metalloprotease abundantly expressed on the outer cell membrane, where it plays roles in several cancer-related processes, including tumor cell proliferation, differentiation, invasion, and angiogenesis [15,16]. Its aberrant overexpression in various malignancies has validated APN as a therapeutic target, with its inhibition demonstrating anti-tumor effects [17]. Bestatin, a classical and clinically utilized inhibitor of APN, is applied as an adjunctive agent in oncology [18,19,20]. Nevertheless, its role to induce differentiation in MM cells, along with the signaling mechanisms involved, is not yet fully explored.

Aberrant activation of the B-cell antigen receptor (BCR) signaling pathway critically contributes to the pathogenesis and progression of MM. Within this pathway, Bruton’s tyrosine kinase (BTK) serves as a pivotal downstream kinase [21] and is instrumental in mediating cellular proliferation, survival, migration, and the development of drug resistance [22,23]. CD79B, a core component of the BCR complex, propagates proliferative and survival signals primarily through activating BTK. Consequently, this signaling axis has gained prominence as a key therapeutic target in hematologic malignancies [24,25]. While studies indicate that APN can influence tumor cell biology via the modulation of specific signaling pathways [16], the precise regulatory relationship and functional interplay between APN and the CD79B/BTK pathway in MM cells remain unexplored.

Building upon this background, our study is designed to investigate how the APN inhibitor Bestatin regulates differentiation in MM cells and to elucidate the associated molecular mechanisms. The specific objectives are to delineate the functional relationship between APN and the CD79B/BTK pathway, pinpoint key downstream transcription factors, and assess the therapeutic potential of combining Bestatin with Ixazomib, a proteasome inhibitor used clinically. This work will unravel the molecular network responsible for Bestatin-induced differentiation in MM, thereby contributing novel theoretical foundations for targeted therapy and offering practical experimental evidence to inform the design of optimized combination regimens.

## 2. Materials and Methods

### 2.1. Main Reagents

Human multiple myeloma (MM) cell lines U266 and RPMI-8226 were procured from the Shanghai Cell Bank (Shanghai, China), and MM1.S cells were obtained from Wuhan Puno Science (Wuhan, China). RPMI-1640 and IMDM cell culture medium were purchased from Gibco (Grand Island, NY, USA). Fetal bovine serum (FBS) was sourced from Shanghai Yikesai Biotech (Shanghai, China). FITC-conjugated anti-human CD49e antibody was acquired from BioLegend (San Diego, CA, USA). Wright–Giemsa staining solution and cell apoptosis detection kits (Annexin V-APC/7-AAD) were supplied by Zhuhai Beso Biotechnology (Zhuhai, China).

### 2.2. Methods

#### 2.2.1. Cell Culture

MM1.S (CL-0614) was kindly provided by Wuhan Procell (Wuhan, China). U266 and RPMI-8226 cell lines are maintained in our laboratory. Cells were cultured in a humidified incubator at 37 °C with 5% CO_2_. MM1.S cells were cultured in RPMI-1640 medium supplemented with 15% FBS. U266 cells in RPMI-1640 medium with 10% FBS, and RPMI-8226 cells in IMDM medium containing 10% FBS.

#### 2.2.2. Flow Cytometry Analysis of APN Expression

Cells (MM1.S, U266, RPMI-8226) in the logarithmic phase were seeded into 6-well plates at a density of 1.5 × 10^5^ cells/mL and cultured for 48 h. Subsequently, cells were harvested by centrifugation, washed once with PBS, and resuspended in 100 µL of PBS. Cell suspensions were incubated with 2 µL of a fluorescently labeled APN-specific antibody for 30 min at 4 °C in the dark. Following incubation, the volume was adjusted to 300 µL with PBS. A minimum of 10,000 events per sample were analyzed using flow cytometry, and data were processed with FlowJo (10.6.2) software.

#### 2.2.3. CCK-8 Assay for Cell Proliferation

Cells were seeded into 96-well plates at a density of 2 × 10^3^ cells per well in 100 µL of complete medium. To minimize evaporation edge effects, the peripheral wells were filled with 100 µL of PBS. After a 2–4 h attachment period, cells were treated with 50 µL of medium containing serial dilutions of the test compounds. Following 48 h of incubation, 10 µL of CCK-8 reagent was added to each well, and the plates were incubated for an additional 1–4 h. The absorbance at 450 nm (OD) was measured using a microplate reader. The cell proliferation inhibition rate was calculated as follows: Inhibition Rate (%) = [(OD_(control)_ − OD_(experimental)_)/OD_(control)_] × 100%. Each condition was assayed in at least three independent replicates.

#### 2.2.4. Wright–Giemsa Staining for Morphological Assessment

To assess differentiation-associated morphological changes, cells were seeded in 6-well plates at 2 × 10^5^ cells/mL (100 µL/well) and treated with various agents for 48 h. Cells were then collected by centrifugation (2500 rpm for 5 min), washed twice with PBS, and used to prepare thin smears on glass slides. After air-drying, the smears were stained with Wright–Giemsa solution according to the manufacturer’s protocol. Cellular morphology was examined and imaged under an oil-immersion light microscope. Cells displaying nuclear condensation, chromatin aggregation, increased cytoplasmic volume, and a reduced nuclear-to-cytoplasmic ratio were classified as differentiated. Differentiation status was recorded, and representative images were captured.

#### 2.2.5. Flow Cytometric Analysis of CD49e Expression

Cells, prepared as single-cell suspensions at 1.5 × 10^5^ cells/mL, were seeded into 6-well plates and exposed to different drug concentrations. Following a 48 h drug treatment, cells were harvested, washed with PBS, and resuspended in 300 µL of pre-cooled PBS. The suspensions were incubated with 2.5 µL of FITC-conjugated anti-CD49e antibody for 30 min at 4 °C in the dark. After washing, the fluorescence intensity of 10,000 cells per sample was analyzed via flow cytometry (FITC channel) (Beckman Coulter, CA, USA). Data were processed and quantified using FlowJo (10.6.2) software.

#### 2.2.6. Western Blot Analysis

Following a 48 h treatment with the indicated drug concentrations, cells (seeded at 1.0 × 10^5^ cells/mL in 6-well plates) were processed for analysis. Briefly, the culture medium was removed, and cells were washed three times with ice-cold PBS. Cell lysis buffer was added to each well, and plates were incubated on ice for 10 min. Lysates were collected, denatured by heating at 99 °C for 10 min, and stored at −40 °C. Following SDS-PAGE separation and protein transfer onto PVDF membranes, nonspecific binding sites were blocked with 5% non-fat milk for 1 h at room temperature. Incubation with primary antibodies (1:1000 dilution) was carried out overnight at 4 °C. After three washes with TBST, membranes were incubated with HRP-conjugated secondary antibodies (1:2000 dilution) for 4 h at 4 °C. Following another three TBST washes, protein bands were visualized using enhanced chemiluminescence (ECL) substrate. Quantification of band intensity was performed using ImageJ (2.0) software.

#### 2.2.7. Quantitative Real-Time PCR (qRT-PCR) Detection

Following a 48 h treatment with various drug concentrations, cells (seeded in 6-well plates at 1.0 × 10^5^ cells/mL) were harvested for RNA extraction using a commercial RNA isolation kit. The extracted RNA was reverse-transcribed into cDNA, which then served as the template for qRT-PCR analysis performed with a fluorescence-based detection kit. The relative mRNA expression levels of target genes were calculated using the 2^−∆∆CT^ method, with GAPDH or β-actin used as internal reference genes.

#### 2.2.8. Apoptosis Assay by Flow Cytometry

Following a 48 h incubation with the indicated drug concentrations, cells (seeded in 6-well plates at 1.0 × 10^5^ cells/mL) were harvested by centrifugation, washed once with PBS, and subsequently resuspended in binding buffer. Apoptosis was then evaluated using an Annexin V-APC and 7-AAD dual-staining kit, adhering to the manufacturer’s protocol. Briefly, cells were incubated with the staining reagents for 30 min at 4 °C in the dark. Flow cytometric analysis was performed immediately after incubation to distinguish and quantify apoptotic cell populations [26].

#### 2.2.9. Senescence-Associated β-Galactosidase (SA-β-Gal) Staining Assay

MM1.S, U266, and RPMI-8226 cells in the log phase were seeded into 6-well plates at a density of 2 × 10^5^ cells/mL and exposed to various concentrations of Bestatin (200, 400, 600, or 800 μM) for 7 days. Following treatment, cells were harvested, washed twice with phosphate-buffered saline (PBS), and fixed with 1× fixative at room temperature for 10–15 min. The fixed cells were then washed twice with PBS. Cells were subsequently incubated overnight at 37 °C without CO_2_ in a freshly prepared SA-β-gal staining solution containing 1 mg/mL X-gal. The next day, SA-β-gal-positive cells (stained blue) were observed under a light microscope.

#### 2.2.10. Immunoglobulin Light Chain ELISA Assay

Log-phase MM1.S and RPMI-8226 cells were adjusted to a density of 1.5 × 10^5^ cells/mL, seeded into 12-well plates, and exposed to varying concentrations of the drug for an additional 48 h. Following incubation, cell culture supernatants were collected and assayed for immunoglobulin light chain levels using an ELISA kit. A standard curve was generated by plotting the optical density (OD) against the corresponding standard concentrations, which was then used to calculate the immunoglobulin light chain concentrations in the samples.

#### 2.2.11. APN Enzyme Activity Assay

Log-phase MM1.S cells were harvested and adjusted to a density of 5 × 10^5^ cells/55 μL, after which 55 μL of the cell suspension was dispensed into each well. Subsequently, 25 μL of diluted Bestatin was added per well to achieve final concentrations of 100, 200, 400, 600, or 800 μM. Following a 5 min incubation at 37 °C, 20 μL of the substrate L-leucine-p-nitroanilide (L9125; Sigma) was added to reach a final concentration of 1.6 mM. Following a 30 min reaction at 37 °C, 70 μL of the supernatant was transferred to a new microplate, and the optical density (OD) at 405 nm was measured using a microplate reader.

#### 2.2.12. EdU Cell Proliferation Assay

Log-phase MM1.S, U266, and RPMI-8226 cells were seeded into 96-well plates at a density of 2 × 10^5^ cells/mL (100 µL/well) and incubated for 24 h. The cells were then treated with Bestatin (200, 400, 600, or 800 µM) or a control drug and cultured for an additional 48 h. An equal volume of 2× EdU working solution was added to achieve a final concentration of 10 µM, followed by a 2 h incubation. Cells were harvested by centrifugation at 2000 rpm for 5 min, fixed with 4% paraformaldehyde for 15 min, and washed three times with wash buffer. After permeabilization with 0.3% Triton X-100 for 10–15 min, cells were washed once or twice. Endogenous peroxidase activity was blocked for 20 min, followed by three washes. The Click reaction solution was prepared according to Supplementary Table S3, and 50 µL was added to each well. The plates were incubated in the dark for 30 min and washed three times. Cells were incubated with Streptavidin-HRP working solution for 30 min and washed three times. TMB chromogenic solution was added and incubated for 5–30 min. The reaction was terminated with 2 M H_2_SO_4_, and the optical density (OD) at 450 nm was measured using a microplate reader.

#### 2.2.13. Bioinformatics Analysis

Publicly available gene expression datasets (GSE6691, GSE10846) and corresponding clinical information were downloaded from the Gene Expression Omnibus (GEO) database. Data processing and analysis were performed using R (version 4.3.1) software. Following probe ID conversion and normalization, samples were stratified into high- and low-expression groups based on the median expression value of CD79B. Kaplan–Meier survival curves were plotted, and the “ggplot2” and “survminer” R packages were utilized for visualization.

#### 2.2.14. Statistical Analysis

All experiments were performed in at least three independent replicates. Data were analyzed using SPSS software and are presented as the mean ± standard error of the mean (SEM). Statistical comparisons between two groups were conducted using a *t*-test, with statistical significance defined as *p* < 0.05.

## 3. Results

### 3.1. The APN Inhibitor Bestatin Promotes Differentiation and Inhibits Proliferation in MM Cells

**Figure 1:**
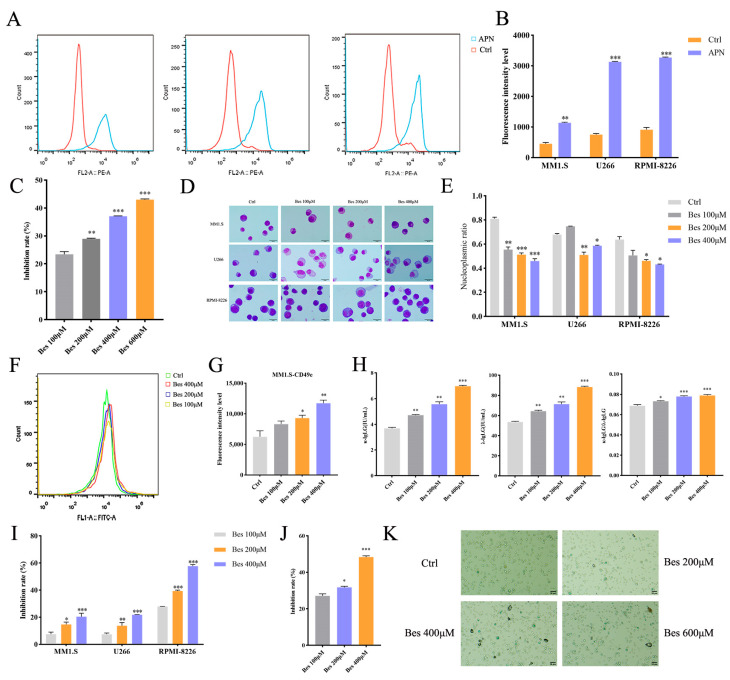
APN inhibitor Bestatin induces differentiation and suppresses proliferation in MM cells. (**A**,**B**) Flow cytometry analysis of APN expression in MM1.S, U266, and RPMI-8226 cells. (**C**) APN enzyme activity assay using MM1.S cells treated with Bestatin. (**D**,**E**) Wright–Giemsa staining images demonstrating concentration-dependent morphological alterations and differentiation hallmarks in MM1.S, U266, and RPMI-8226 cells after 48 h treatment with Bestatin. (**F**,**G**) Flow cytometry analysis showing the upregulation of CD49e on MM1.S cells following 48 h exposure to graded concentrations of Bestatin. (**H**) ELISA detection of κ-IgLG, λ-IgLG levels and κ/λ ratio in MM1.S cells treated with Bestatin for 48 h. (**I**) CCK-8 viability assay of MM1.S, U266, and RPMI-8226 cells treated with different doses of Bestatin for 48 h. (**J**) EdU assay to detect the proliferation of MM1.S cells treated with Bestatin for 24 h. (**K**) SA-β-gal staining to detect cell senescence in MM1.S cells treated with Bestatin for 7 days. Data are presented as mean ± SEM. Statistical significance compared to the control group is indicated as follows: * p < 0.05, ** p < 0.01, *** p < 0.001.

Initial analysis of three MM cell lines (MM1.S, U266, RPMI-8226) by flow cytometry revealed consistently high expression of APN (Figure 1A,B), implicating its potential functional relevance in MM. We initially assessed the inhibitory effect of Bestatin on APN enzymatic activity, and the results demonstrated that it suppressed APN activity in a concentration-dependent manner (Figure 1C). We next evaluated the effect of Bestatin on cellular differentiation using morphological and immunophenotypic assessments. Wright–Giemsa staining of cells treated with 400, 600, and 800 µM of Bestatin demonstrated classic hallmarks of differentiation across all lines, including nuclear condensation, chromatin aggregation, increased cytoplasmic volume, and a decreased nuclear-to-cytoplasmic ratio (Figure 1D,E). Furthermore, flow cytometry indicated a dose-dependent upregulation of the differentiation marker CD49e on MM1.S cells following Bestatin treatment (Figure 1F,G). To further functionally validate the differentiation, the secretion levels of κ-IgLG and λ-IgLG in the cell culture supernatants were measured by ELISA. Bestatin treatment significantly increased the secretion of both κ-IgLG and λ-IgLG and elevated the κ/λ ratio, indicating that the cells differentiated into functional plasma cells (Figure 1H). As differentiation is typically coupled to reduced proliferative potential, we assessed cell viability using the CCK-8 assay. Bestatin treatment resulted in a pronounced, concentration-dependent suppression of viability in all three MM cell lines (Figure 1I). Concurrently, the EdU proliferation assay further demonstrated that Bestatin significantly reduced DNA synthesis in MM cells and inhibited their proliferation (Figure 1J), confirming its antiproliferative effect. Additionally, we evaluated cellular senescence using SA-β-gal staining, which showed that the proportion of positively stained senescent cells increased significantly with escalating concentrations of Bestatin (Figure 1K). Taken together, these data demonstrate that Bestatin concurrently induces differentiation and inhibits proliferation in MM cells.

### 3.2. Bestatin Promotes MM Cell Differentiation via the CD79B/BTK Pathway

To delineate the signaling mechanism by which Bestatin induces differentiation, we employed an integrated bioinformatics strategy. First, RNA sequencing of Bestatin-treated MM1.S cells was performed to profile differentially expressed genes (Supplementary Figure S1A). In parallel, we analyzed the publicly available GSE6691 dataset from the GEO database, identifying genes differentially expressed between MM samples and normal bone marrow lymphocyte (NBL) controls (Supplementary Figure S1B). The intersection of these two independent gene sets yielded 149 overlapping candidates potentially linked to differentiation (Supplementary Figure S1C). A protein–protein interaction (PPI) network constructed from these genes facilitated the prioritization of hub genes (Supplementary Figure S1D). Subsequent correlation analysis revealed a significant negative association between the expression of APN and the key B-cell receptor components CD79B and BTK (Supplementary Figure S1E). Furthermore, survival analysis demonstrated that MM patients exhibiting high CD79B/BTK expression had a significantly poorer overall survival compared to the low-expression group (Supplementary Figure S1F). These bioinformatics findings collectively indicated that CD79B and BTK are critically linked to APN expression and patient prognosis, thereby prioritizing them for further functional investigation.

**Figure 2:**
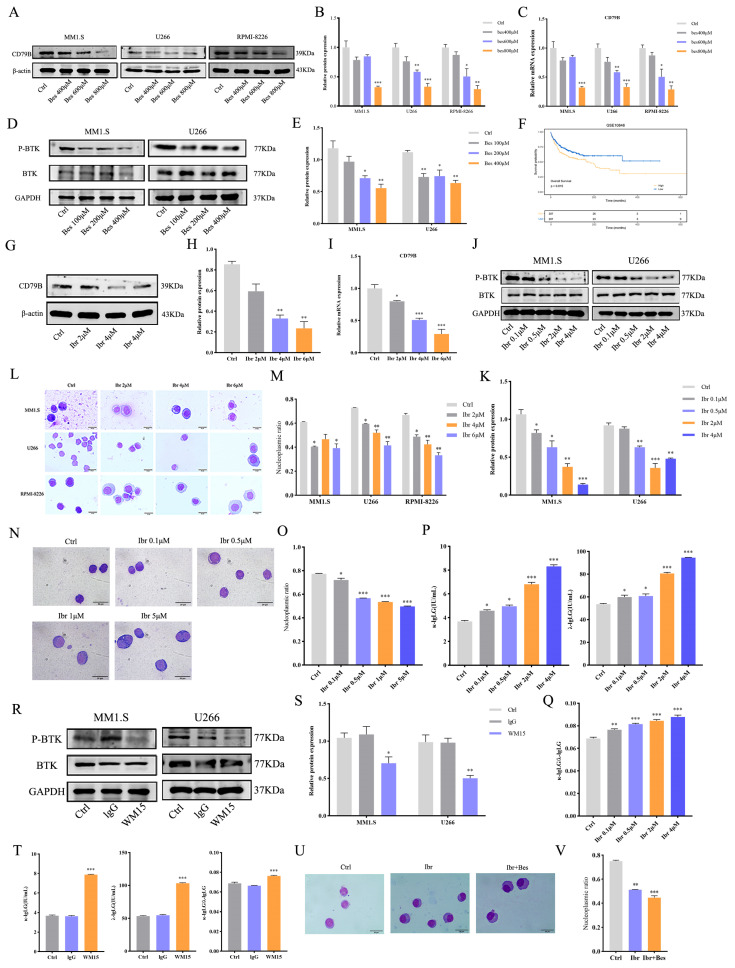
Bestatin induces MM cell differentiation via the CD79B/BTK pathway. (**A**,**B**) Western blot analysis of CD79B protein expression in MM1.S, U266, and RPMI-8226 cells treated with Bestatin for 48 h. (**C**) qRT-PCR analysis of CD79B mRNA expression in MM1.S, U266, and RPMI-8226 cells treated with Bestatin for 48 h. (**D**,**E**) Western blot analysis of BTK and p-BTK protein expression in MM1.S and U266 cells treated with Bestatin for 12 h. (**F**) Kaplan–Meier survival analysis of MM patients stratified by CD79B expression based on the GEO dataset GSE10846. (**G**,**H**) Western blot analysis of CD79B protein expression in MM1.S cells treated with Ibrutinib for 48 h. (**I**) qRT-PCR analysis of CD79B mRNA expression in MM1.S cells treated with Ibrutinib for 48 h. (**J**,**K**) Western blot analysis of BTK and p-BTK protein expression in MM1.S and U266 cells treated with Ibrutinib for 6 h. (**L**,**M**) Wright–Giemsa staining to observe morphological differentiation of MM1.S, U266, and RPMI-8226 cells treated with Ibrutinib for 48 h. (**N**,**O**) Wright–Giemsa staining to observe plasma cell-like differentiation of MM1.S cells treated with Ibrutinib for 7 days. (**P**,**Q**) ELISA detection of κ-IgLG and λ-IgLG levels in MM1.S cells treated with Ibrutinib for 48 h. (**R**,**S**) Western blot analysis of BTK and p-BTK expression in MM1.S and U266 cells treated with APN-neutralizing antibody WM15 for 12 h. (**T**) ELISA detection of κ-IgLG and λ-IgLG levels in MM1.S cells treated with WM15 for 48 h. (**U**,**V**) Wright–Giemsa staining to observe differentiation of MM1.S cells treated with Ibrutinib alone or combined with Bestatin for 48 h. Data are presented as mean ± SEM. Statistical significance compared to the control group is indicated as follows: * p < 0.05, ** p < 0.01, *** p < 0.001.

To investigate whether CD79B and BTK function as downstream effectors of APN inhibition, Western blot analysis was performed to examine CD79B expression in MM1.S, U266, and RPMI-8226 cells following treatment with 400, 600, and 800 µM of Bestatin. As the concentration of Bestatin increased, a dose-dependent downregulation of CD79B protein was observed (Figure 2A,B). This reduction was also evident at the transcriptional level, as qRT-PCR confirmed a significant decrease in CD79B mRNA under identical conditions (Figure 2C). Subsequently, MM1.S and U266 cells were treated with 100, 200, or 400 µM of Bestatin. p-BTK protein expression decreased significantly as the Bestatin concentration increased (Figure 2D,E). Additionally, analysis of the independent GSE10846 dataset demonstrated that high CD79B expression is associated with significantly increased mortality in MM patients (Figure 2F), reinforcing its adverse prognostic role and aligning with the pathway’s functional importance.

To clarify whether inhibition of CD79B/BTK is sufficient to drive differentiation, rather than being merely regulated by Bestatin, we employed the selective inhibitor Ibrutinib. Treatment of MM1.S cells with 2, 4, and 6 µM of Ibrutinib led to a dose-dependent decrease in CD79B protein levels, as confirmed by Western blot (Figure 2G,H). Furthermore, treatment of MM1.S cells with 2, 4, and 6 µM of Ibrutinib led to a dose-dependent decrease in CD79B mRNA levels, as confirmed by qRT-PCR (Figure 2I). Subsequently, MM1.S and U266 cells were treated with 0.1, 0.5, 2, or 4 µM of Ibrutinib. Western blot analysis also showed that p-BTK protein expression decreased significantly as the Ibrutinib concentration increased (Figure 2J,K). Morphological analysis via Wright–Giemsa staining demonstrated that Ibrutinib treatment alone induced classic differentiation features, including nuclear condensation, chromatin aggregation, increased cytoplasmic volume, and a reduced nuclear-to-cytoplasmic ratio, in a concentration-dependent manner (Figure 2L,M). To further exclude off-target effects associated with high Ibrutinib concentrations, a low-concentration, prolonged-exposure experiment was conducted. Treating MM1.S cells with 0.1 μM of Ibrutinib for 7 days induced typical plasma cell-like differentiation morphology (Figure 2N,O), confirming that Ibrutinib induces differentiation by targeting BTK rather than through nonspecific toxicity. Concurrently, ELISA results showed that Ibrutinib treatment significantly increased the secretion levels of κ-IgLG and λ-IgLG and elevated the κ/λ ratio, confirming from a functional perspective that it promotes the differentiation of MM cells into functional plasma cells (Figure 2P,Q). This result indicates that suppressing the CD79B/BTK axis recapitulates the differentiation-inducing effect of Bestatin, supporting its functional involvement in the differentiation cascade.

To exclude off-target effects of small-molecule inhibitors, we used the APN-neutralizing antibody WM15 for validation. WM15 significantly downregulated p-BTK expression (Figure 2R,S) and promoted immunoglobulin light chain secretion (Figure 2T), yielding effects similar to those of Bestatin. Furthermore, combining Ibrutinib with Bestatin yielded a more pronounced differentiation compared to Ibrutinib alone (Figure 2U,V). Collectively, these functional experiments establish that CD79B and BTK are key downstream mediators of APN and mediate the pro-differentiation effects of Bestatin.

### 3.3. The CD79B/BTK Pathway Drives MM Cell Differentiation via the Transcription Factor STAT3

**Figure 3:**
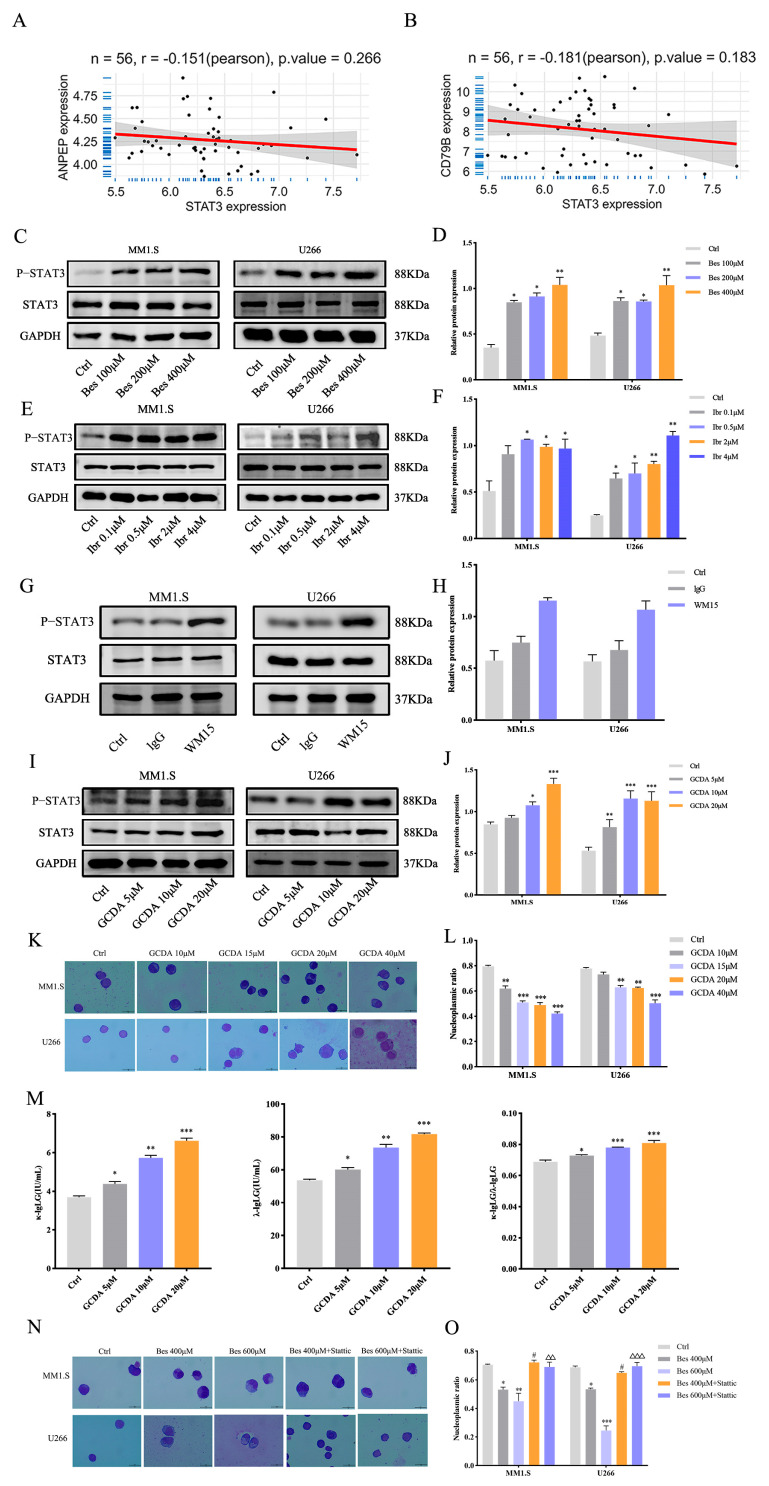
STAT3 drives differentiation and acts downstream of the CD79B/BTK pathway in MM cells. (**A**,**B**) Bioinformatics analysis of the correlation between APN (**A**) or CD79B (**B**) and STAT3 expression. (**C**,**D**) Western blot analysis of p-STAT3 protein expression in MM1.S and U266 cells treated with Bestatin for 12 h. (**E**,**F**) Western blot analysis of p-STAT3 protein expression in MM1.S and U266 cells treated with Ibrutinib for 6 h. (**G**,**H**) Western blot analysis of p-STAT3 expression in MM1.S and U266 cells treated with WM15 for 12 h. (**I**,**J**) Western blot analysis of p-STAT3 protein expression in MM1.S and U266 cells treated with GCDA for 6 h. (**K**,**L**) Wright–Giemsa staining to observe morphological differentiation of MM1.S and U266 cells treated with GCDA for 48 h. (**M**) ELISA detection of κ-IgLG and λ-IgLG levels in MM1.S cells treated with GCDA for 48 h. (**N**,**O**) Wright–Giemsa staining to observe morphological changes in MM1.S and U266 cells treated with Bestatin alone or combined with Stattic for 48 h. Data are presented as mean ± SEM. Significance is denoted as: * p < 0.05, ** p < 0.01, *** p < 0.001 vs. control; *# p* < 0.05 vs. same concentration of Bestatin alone; △△ *p* < 0.01, △△△ *p* < 0.001 vs. same concentration of Bestatin alone.

Transcription factors serve as central regulators directing MM cell differentiation. To pinpoint the specific factor through which the CD79B/BTK axis exerts its effect, we conducted a targeted search among transcription factors that may affect differentiation, including XBP1, BCL6, IRF4, STAT3, FOSL1, BATF, and others. Given that Bestatin induces differentiation, we hypothesized that it likely upregulates a pro-differentiation transcription factor. Furthermore, since Bestatin suppresses the CD79B/BTK pathway, this candidate factor was expected to be inversely correlated with APN and CD79B expression. To test this hypothesis, bioinformatics analyses were performed to assess correlations between APN/CD79B and the candidate factors (Supplementary Tables S1 and S2). This analysis identified STAT3 as the sole candidate exhibiting a significant negative correlation with both APN and CD79B (Figure 3A,B), suggesting its potential role as a downstream effector in the APN-CD79B/BTK axis. Thus, we propose that STAT3 is a critical transcription factor mediating CD79B/BTK-regulated differentiation in MM cells.

To delineate the hierarchical relationship between the CD79B/BTK pathway and STAT3, the specific inhibitor Ibrutinib was deployed. Our premise was that if STAT3 acts downstream of CD79B/BTK, then inhibiting the pathway should mirror the effect of Bestatin and lead to STAT3 upregulation. Consistent with this expectation, MM1.S and U266 cells were treated with Bestatin for 12 h, and STAT3 phosphorylation levels were assessed via Western blot. The results indicated that STAT3 phosphorylation levels increased significantly in both cell lines with escalating Bestatin concentrations (Figure 3C,D). Ibrutinib treatment similarly upregulated STAT3 phosphorylation levels (Figure 3E,F). To exclude off-target effects of small-molecule inhibitors, the APN-neutralizing antibody WM15 was used for validation, which also significantly upregulated p-STAT3 levels (Figure 3G,H). This finding provides direct functional evidence that STAT3 is a downstream transcriptional target of the CD79B/BTK pathway, thereby solidifying its position within the APN-CD79B/BTK-STAT3 signaling axis.

To determine whether STAT3 activation is sufficient to trigger differentiation, MM1.S and U266 cells were treated with the specific STAT3 agonist GCDA. As expected, Western blot analysis showed that GCDA significantly upregulated STAT3 phosphorylation levels (Figure 3I,J). Morphological assessment by Wright–Giemsa staining revealed that GCDA-treated cells displayed classic hallmarks of differentiation—including nuclear condensation, chromatin aggregation, expanded cytoplasm, and a reduced nuclear-to-cytoplasmic ratio—as observed by Wright–Giemsa staining (Figure 3K,L). Concurrently, ELISA results showed that GCDA significantly promoted the secretion of κ-IgLG and λ-IgLG and elevated the κ/λ ratio, confirming functional plasma cell differentiation (Figure 3M). These data confirm that direct STAT3 activation is sufficient to drive MM cell differentiation. Next, to determine whether STAT3 is necessary for Bestatin-induced differentiation, cotreatment experiments were conducted using Bestatin alone or in combination with the STAT3 inhibitor Stattic. Wright–Giemsa staining revealed that Stattic significantly attenuated the differentiation-associated morphological changes induced by Bestatin in both MM1.S and U266 cells (Figure 3N,O). Taken together, these experiments demonstrate that STAT3 activation is both sufficient and essential for Bestatin-mediated differentiation. These findings conclusively establish STAT3 as the pivotal downstream transcription factor of the CD79B/BTK pathway, essential for executing the differentiation in MM cells.

### 3.4. Bestatin Synergizes with IL-6 to Promote Differentiation and Inhibit Proliferation in MM Cells

**Figure 4:**
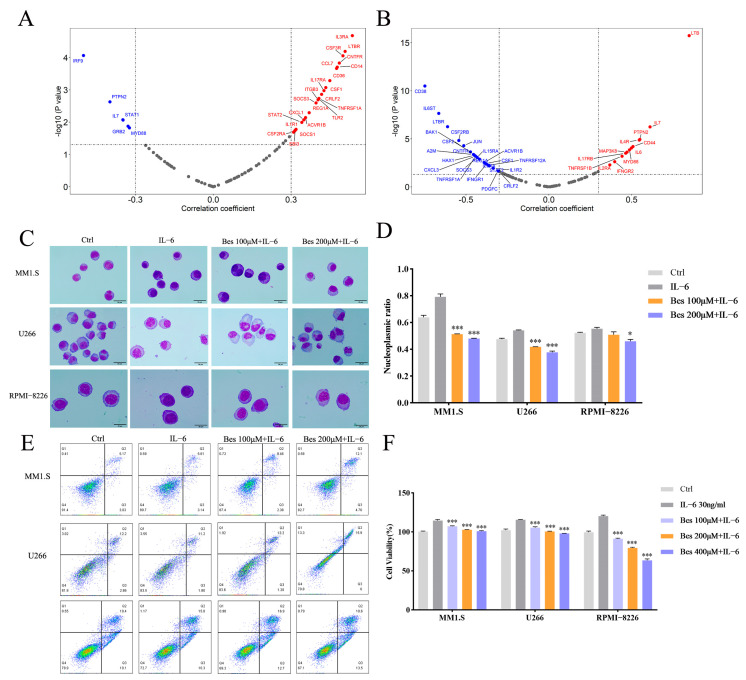
Combined treatment with IL-6 and Bestatin promotes differentiation and suppresses proliferation of MM cells. (**A**,**B**) Bioinformatic analysis of correlations between the expression of APN (**A**) or CD79B (**B**) and IL-6-STAT3 pathway-related genes. (**C**,**D**) Wright–Giemsa staining depicting the morphological differentiation of MM1.S, U266, and RPMI-8226 cells after 48 h treatment under the following conditions: untreated control (Ctrl), IL-6 alone, 100 µM of Bestatin + IL-6, and 200 µM of Bestatin + IL-6. (**E**) Apoptosis of MM1.S, U266, and RPMI-8226 cells, as quantified by flow cytometry using Annexin V-APC/7-AAD staining, following the indicated 48 h treatments. (**F**) Cell proliferation of MM1.S, U266, and RPMI-8226 cells, evaluated by CCK-8 assay, after 48 h exposure to the specified treatments. Data are presented as mean ± SEM. Statistical significance versus the IL-6 monotherapy group is indicated as * p < 0.05, *** p < 0.001.

The IL-6-STAT3 signaling axis is a well-characterized pathway that remains constitutively active in MM cells [27,28,29]. Based on our findings of STAT3 as a critical driver of MM cell differentiation, we postulated that Bestatin and the canonical STAT3 activator IL-6 might act cooperatively, potentially yielding synergistic effects to promote differentiation and inhibit proliferation. To explore this hypothesis, bioinformatic correlation analysis revealed robust positive associations between APN, CD79B, and core components of the IL-6-STAT3 pathway (Figure 4A,B), providing a rationale for functional synergy. Subsequently, MM1.S, U266, and RPMI-8226 cells were treated with 100 or 200 µM of Bestatin in the presence of IL-6 for 48 h. Morphological assessment via Wright–Giemsa staining confirmed that the combination robustly induced differentiation across all cell lines (Figure 4C,D). This pro-differentiation effect was accompanied by a significant induction of apoptosis in cells treated with Bestatin and IL-6 compared to either agent alone, as measured by flow cytometry (Figure 4E). Correspondingly, CCK-8 assays demonstrated a marked reduction in cell viability upon co-treatment compared to single agents (Figure 4F). Together, these results demonstrate that Bestatin and IL-6 act synergistically to promote differentiation, induce apoptotic cell death, and inhibit proliferation in MM cells, likely through convergent activation of the STAT3 pathway.

### 3.5. Both CD79B/BTK Inhibitors and STAT3 Agonists Sensitize MM Cells to Ixazomib

**Figure 5:**
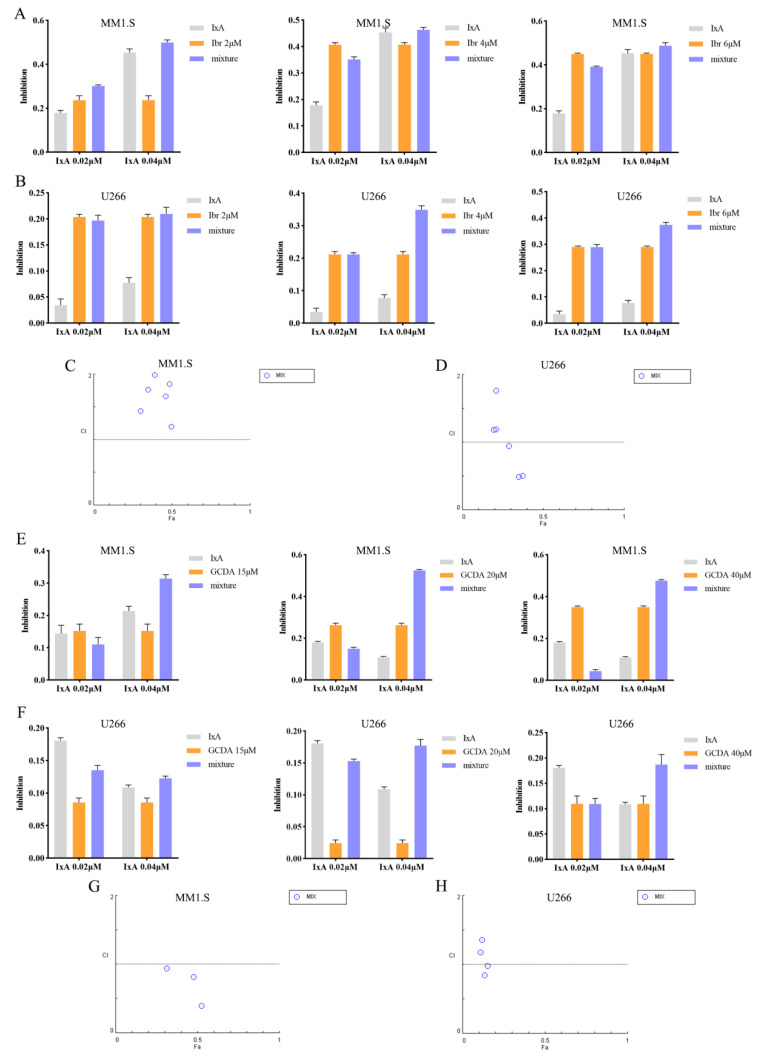
The CD79B/BTK inhibitor Ibrutinib and the STAT3 agonist GCDA potentiate the antitumor activity of Ixazomib. (**A**,**B**) Cell viability inhibition rates of MM1.S (**A**) and U266 (**B**) cells treated for 48 h with the indicated combinations of Ibrutinib and Ixazomib, as determined by CCK-8 assay. (**C**,**D**) Combination Index (CI) analysis for Ibrutinib and Ixazomib in MM1.S (**C**) and U266 (**D**) cells, calculated using Compusyn software. CI < 1 indicates synergy. (**E**,**F**) Inhibition of cell viability, measured by CCK-8 assay, in MM1.S (**E**) and U266 (**F**) cells following 48 h co-treatment with GCDA and Ixazomib. (**G**,**H**) CI analysis for GCDA and Ixazomib in MM1.S (**G**) and U266 (**H**) cells, analyzed with Compusyn software.

Cellular differentiation is known to enhance chemosensitivity [30,31]. Building on our findings that CD79B/BTK inhibition and STAT3 activation drive MM cell differentiation, we investigated whether modulating this pathway could potentiate the efficacy of the clinical proteasome inhibitor Ixazomib. To evaluate potential synergistic anti-tumor effects, the CD79B/BTK inhibitor Ibrutinib and the STAT3 agonist GCDA were individually combined with Ixazomib—a clinically approved agent for MM treatment—and assessed in vitro. MM1.S and U266 cells were treated with varying concentrations of Ibrutinib (2, 4, and 6 µM) in combination with Ixazomib (0.02 and 0.04 µM). Cell viability was determined using the CCK-8 assay, and the combination index (CI) was calculated via Compusyn (version 1.0) software (CI < 1 indicates synergy). Co-treatment resulted in a concentration-dependent enhancement of cytotoxicity beyond the effect of either agent alone (Figure 5A,B). Compusyn analysis revealed synergistic interactions (CI < 1) in MM1.S cells at Ibrutinib–Ixazomib combinations of 2 µM + 0.02 µM, 2 µM + 0.04 µM, 4 µM + 0.04 µM, and 6 µM + 0.04 µM. In U266 cells, synergism was observed at 2 µM + 0.04 µM, 4 µM + 0.04 µM, and 6 µM + 0.04 µM (Figure 5C,D), indicating that CD79B/BTK pathway inhibition synergizes with Ixazomib to augment anti-tumor activity.

Similarly, GCDA (15, 20, and 40 µM) was combined with Ixazomib (0.02 and 0.04 µM) in MM1.S and U266 cells. Cell viability and CI values were assessed using identical methods. The results showed that co-treatment led to a concentration-dependent increase in cytotoxicity compared to single-agent treatments (Figure 5E,F). Synergistic effects (CI < 1) were observed at GCDA-Ixazomib combinations of 15 µM + 0.02 µM, 20 µM + 0.04 µM, and 40 µM + 0.04 µM in both MM1.S and U266 cell lines (Figure 5G,H), suggesting that STAT3 activation-mediated differentiation synergistically enhances the anti-tumor efficacy of Ixazomib. In summary, both the CD79B/BTK inhibitor Ibrutinib and the STAT3 agonist GCDA can potentiate the anti-tumor activity of Ixazomib by promoting MM cell differentiation, with significant synergistic effects observed at specific concentration combinations.

### 3.6. The APN Inhibitor Bestatin Enhances the Chemosensitivity of MM Cells to Ixazomib

**Figure 6:**
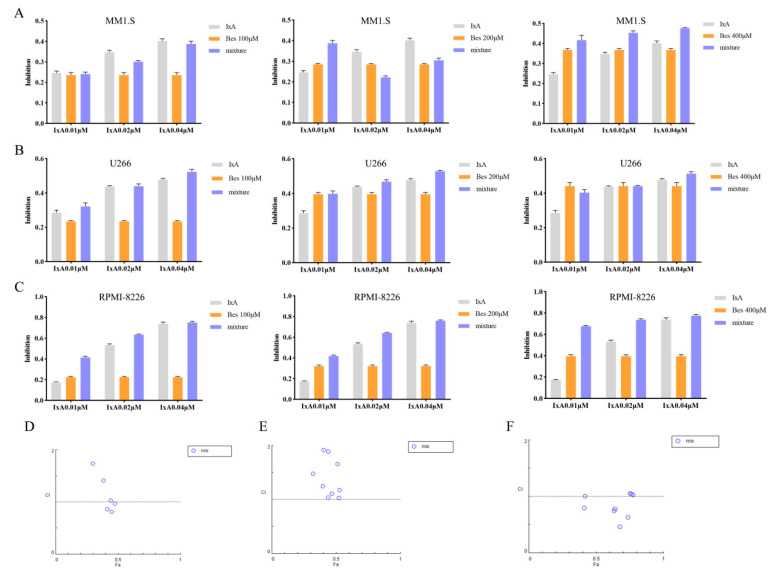
Synergistic Inhibition of MM Cells by Bestatin in Combination with Ixazomib (**A**–**C**). The inhibitory effect on cell viability was evaluated by CCK-8 assay in MM1.S (**A**), U266 (**B**), and RPMI-8226 (**C**) cells following 48 h co-treatment with Bestatin and Ixazomib at the indicated concentrations. (**D**–**F**) Synergy analysis for Bestatin and Ixazomib in MM1.S (**D**), U266 (**E**), and RPMI-8226 (**F**) cells, as quantified by the CI using Compusyn software. CI < 1 indicates a synergistic interaction.

Given our prior observation that the APN inhibitor Bestatin induces MM cell differentiation, we postulated that this pro-differentiation effect might enhance sensitivity to the clinical agent Ixazomib, thereby achieving a synergistic anti-tumor effect through combined differentiation therapy and chemotherapy. To test this, MM1.S, U266, and RPMI-8226 cells were treated with combinations of Bestatin (100, 200, and 400 µM) and Ixazomib (0.01, 0.02, and 0.04 µM). CCK-8 assays revealed that Bestatin potentiated the cytotoxicity of Ixazomib in a clear dose-dependent manner across all cell lines (Figure 6A,C). Compusyn analysis (CI < 1) identified synergistic interactions: for MM1.S cells at 200 µM + 0.04 µM, 400 µM + 0.02 µM, and 400 µM + 0.04 µM; for U266 cells at 200 µM + 0.04 µM and 400 µM + 0.04 µM; and for RPMI-8226 cells at 100 µM + 0.02 µM, 200 µM + 0.02 µM, and 400 µM + 0.02 µM (Figure 6D,F). These results demonstrate that the combination of Bestatin and Ixazomib exerts a synergistic anti-tumor effect. The finding that Bestatin-mediated differentiation chemosensitizes MM cells provides direct experimental support for the therapeutic potential of combining differentiation inducers with conventional chemotherapy.

## 4. Discussion

This work elucidates, for the first time, the molecular mechanism by which the APN inhibitor Bestatin induces differentiation in MM cells, identifying the CD79B/BTK-STAT3 axis as the critical signaling pathway. This finding proposes a novel therapeutic strategy for MM. We initially observed a robust overexpression of APN across three representative MM cell lines (MM1.S, U266, and RPMI-8226), providing a mechanistic rationale for targeting APN in this malignancy. Although Bestatin is a clinically used APN inhibitor [18], its specific biological impact on MM cells has remained elusive. Our results clearly define its dual role in simultaneously promoting differentiation and inhibiting proliferation of MM cells. This not only fills a fundamental knowledge gap regarding Bestatin’s mode of action in MM but also provides compelling experimental support for its clinical repurposing.

At the molecular level, the CD79B/BTK signaling axis serves as a pivotal regulator controlling the proliferation, survival, and differentiation of MM cells. Its aberrant activation constitutes a key driver of disease progression. As an essential component of B-cell receptor signaling, sustained activity of CD79B/BTK transmits persistent proliferative and pro-survival signals [24,25]. Supporting its clinical relevance, our analysis of patient samples revealed that high expression of CD79B/BTK is significantly associated with shortened overall survival in MM. This correlation suggests that this pathway likely fuels malignant progression by preserving tumor cells in an undifferentiated, highly proliferative state. Mechanistically, we delineated that Bestatin attenuates the protein and mRNA expression of both CD79B and BTK in a dose-dependent manner across MM1.S, U266, and RPMI-8226 cell lines, coinciding with the induction of differentiation. Crucially, the specific CD79B/BTK inhibitor Ibrutinib alone was sufficient to recapitulate a concentration-dependent, differentiation-promoting effect analogous to Bestatin. These convergent results robustly affirm that inhibition of the CD79B/BTK pathway is a critical trigger for MM cell differentiation. It is noteworthy that in other B-cell malignancies, such as diffuse large B-cell lymphoma (DLBCL), the core function of this pathway centers on driving proliferation, primarily through NF-κB-mediated cell cycle progression [32]. In contrast, our findings in MM uncover a distinct functional paradigm: while CD79B/BTK signaling contributes to cell survival and proliferation, its most prominent and consequential role upon inhibition is the potent induction of terminal differentiation. Thus, within the MM context, the promotion of differentiation emerges as the paramount biological function regulated by this pathway.

This study further delineates STAT3 as a pivotal transcription factor orchestrating the differentiation of MM cells. This role aligns with and extends the documented pro-differentiation function of STAT3 across diverse cellular contexts [33,34,35], highlighting its regulatory capacity. Our mechanistic investigation pinpointed STAT3 as a critical downstream responsive element of the CD79B/BTK pathway, where its activation is both necessary and sufficient to drive MM cell differentiation. Empirical evidence solidified this centrality: The STAT3 agonist GCDA alone is sufficient to trigger differentiation, whereas the inhibitor Stattic effectively abrogates the differentiation induced by Bestatin, solidifying the essential role of STAT3 in this process. The function of STAT3 we identified exhibits remarkable consistency with its actions in other lineages. In myeloid progenitors, STAT3 activation directs neutrophilic differentiation, a process potentiated by G-CSF or STAT3 overexpression [33]. In neuronal development, STAT3 facilitates maturation by directly regulating the transcription of the Sox6 gene [34]. Most pertinently, STAT3 is a known regulator of the terminal differentiation of normal B cells into antibody-secreting plasma cells [35]. Herein, we demonstrate that Bestatin, via suppressing the CD79B/BTK axis, subsequently activates STAT3. This cascade effectively reinstates a latent, physiological differentiation program in MM cells, thereby revealing an intrinsic conceptual link between targeting MM and harnessing the body’s natural B-cell maturation machinery for therapeutic benefit.

The differentiation state of tumor cells critically influences their sensitivity to chemotherapy, making differentiation induction a promising strategy to potentiate treatment efficacy. This principle is exemplified in acute promyelocytic leukemia, where combining differentiation agents like all-trans retinoic acid with chemotherapy can achieve curative results [14]. Anchored in this rationale, our study demonstrates that modulating the APN-CD79B/BTK-STAT3 axis synergistically enhances Ixazomib-based chemotherapy in MM. Specifically, Bestatin potentiated the cytotoxicity of Ixazomib in a concentration-dependent manner, with defined combinations yielding significant synergy. This effect was pathway-specific, as the CD79B/BTK inhibitor Ibrutinib and the STAT3 agonist GCDA similarly augmented Ixazomib’s activity by promoting differentiation. These findings carry direct translational implications. While the proteasome inhibitor Ixazomib is a mainstay for treating relapsed/refractory MM, the emergence of drug resistance constrains its long-term utility. Our work confirms that by inducing differentiation via the APN-CD79B/BTK-STAT3 pathway, resensitizes MM cells to Ixazomib, thereby proposing a novel strategy to circumvent resistance. Bestatin offers a particularly viable combination partner with Ixazomib due to its established safety profile from prior clinical use [18], which could expedite clinical translation. Furthermore, the synergistic activity observed with alternative pathway modulators like Ibrutinib and GCDA introduces flexible therapeutic alternatives, allowing regimen tailoring for patients with differing tolerances or response profiles.

## 5. Conclusions

In summary, this study establishes that the APN inhibitor Bestatin induces differentiation in MM cells by targeting the CD79B/BTK-STAT3 signaling axis. Within this pathway, CD79B/BTK serves as an upstream inhibitory node, whereas STAT3 functions as a downstream transcriptional activator; their coordinated regulation is essential for driving the differentiation program. Furthermore, Bestatin synergizes with the proteasome inhibitor Ixazomib to enhance cytotoxic effects. This synergistic potential is pathway-mediated, as evidenced by the ability of the BTK inhibitor Ibrutinib and the STAT3 agonist GCDA to similarly potentiate Ixazomib’s activity. These findings address a significant gap in understanding how APN inhibitors exert antitumor effects in MM. They provide a mechanistic foundation for a novel “differentiation induction plus chemotherapy” strategy. Further investigation using in vivo models and clinical studies is warranted to validate the translational potential of this combinatorial approach.

## Data Availability

The original contributions presented in this study are included in the article/Supplementary Materials. Further inquiries can be directed to the corresponding author.

## Supplementary Materials

The following supporting information can be downloaded at: https://www.mdpi.com/article/10.3390/cells15100949/s1, Figure S1: Bioinformatics analysis identifying CD79B/BTK as key downstream targets of APN in multiple myeloma. (A) Volcano plot of differentially expressed genes from RNA sequencing of Bestatin-treated MM1.S cells. (B) Volcano plot of differentially expressed genes from the GSE6691 dataset comparing MM samples and normal bone marrow lymphocytes (NBL). (C) Venn diagram showing 149 overlapping candidate genes potentially associated with differentiation. (D) Protein-protein interaction (PPI) network of the overlapping genes for hub gene identification. (E) Correlation analysis between APN expression and CD79B/BTK expression. (F) Kaplan-Meier survival analysis of MM patients stratified by CD79B/BTK expression levels. Figure S2: Ibrutinib treatment inhibited BTK phosphorylation and upregulated STAT3 phosphorylation in MM1.S and U266 cells (three independent replicates). Figure S3: GCDA treatment promoted STAT3 phosphorylation in MM1.S and U266 cells (three independent replicates). Figure S4: Wright-Giemsa staining to observe morphological differentiation of MM1.S cell treated with Ibrutinib for 48 h. Figure S5: (A) CCK-8 viability assay of MM1.S cell treated with different doses of Bestatin for 24 h; (B) The apoptosis rate of MM1.S cells treated with Bestatin was detected by flow cytometry. Table S1: Correlation plot—APN transcription factor; Table S2: Correlation plot—CD79B transcription factor. Table S3: Preparation of Click Reaction Mixture.

## Abbreviations

The following abbreviations are used in this manuscript:

APN	Aminopeptidase N
MM	Multiple myeloma
BCR	B Cell Antigen Receptor
BTK	Bruton’s Tyrosine Kinase
STAT3	Signal Transducer and Activator of Transcription 3
GCDA	Glycochenodeoxycholic Acid
IL-6	Interleukin-6
