# Supplementary material for: APN Inhibitor Bestatin Induces MM Cell Differentiation Through the CD79B/BTK/STAT3 Pathway
Source: Cells. 2026 May 21;15(10):949. doi: 10.3390/cells15100949 (PMC13204620; doi:10.3390/cells15100949)
Supplement: Supplementary file 1 [file cells-15-00949-s001.zip › Describtion.pdf]

Supplementary Figure S1A：RNA sequencing was performed on Bestatin-treated MM1.S cells to analyze differentially expressed genes ；Supplementary Figure S1B：Analyzed the publicly available dataset GSE6691 from the GEO database, identifying differentially expressed genes between multiple myeloma (MM) samples and normal bone marrow lymphocytes (NBL) controls；Supplementary Figure S1C：The intersection of these two independent gene sets yielded 149 overlapping candidate genes potentially associated with differentiation；Supplementary Figure S1D：A protein-protein interaction (PPI) network constructed from these genes facilitated the identification of core genes；Supplementary Figure S1E： Subsequent correlation analysis revealed that APN expression showed significant positive correlations with the expression of key B-cell receptor components CD79B and BTK ；Supplementary Figure S1F：Survival analysis indicated that multiple myeloma patients with high CD79B/BTK expression exhibited significantly shorter overall survival compared to the low-expression group.

Supplementary Figure S2: Ibrutinib treatment inhibited BTK phosphorylation and upregulated STAT3 phosphorylation in MM1.S and U266 cells (three independent replicates).

Supplementary Figure S3: GCDA treatment promoted STAT3 phosphorylation in MM1.S and U266 cells (three independent replicates).

Supplementary Figure S4: Wright-Giemsa staining to observe morphological differentiation of MM1.S cell treated with Ibrutinib for 48 h.

Supplementary Figure S5A:CCK-8 viability assay of MM1.S cell treated with different doses of Bestatin for 24 h ;Supplementary Figure S5B:The apoptosis rate of MM1.S cells treated with Bestatin was detected by flow cytometry.

Supplementary Tables S1 and S2：Bioinformatics analysis was performed to assess correlations between APN/CD79B and candidate factors .
