# Supplementary figures and images for: APN Inhibitor Bestatin Induces MM Cell Differentiation Through the CD79B/BTK/STAT3 Pathway
Source: Cells. 2026 May 21;15(10):949. doi: 10.3390/cells15100949 (PMC13204620; doi:10.3390/cells15100949)

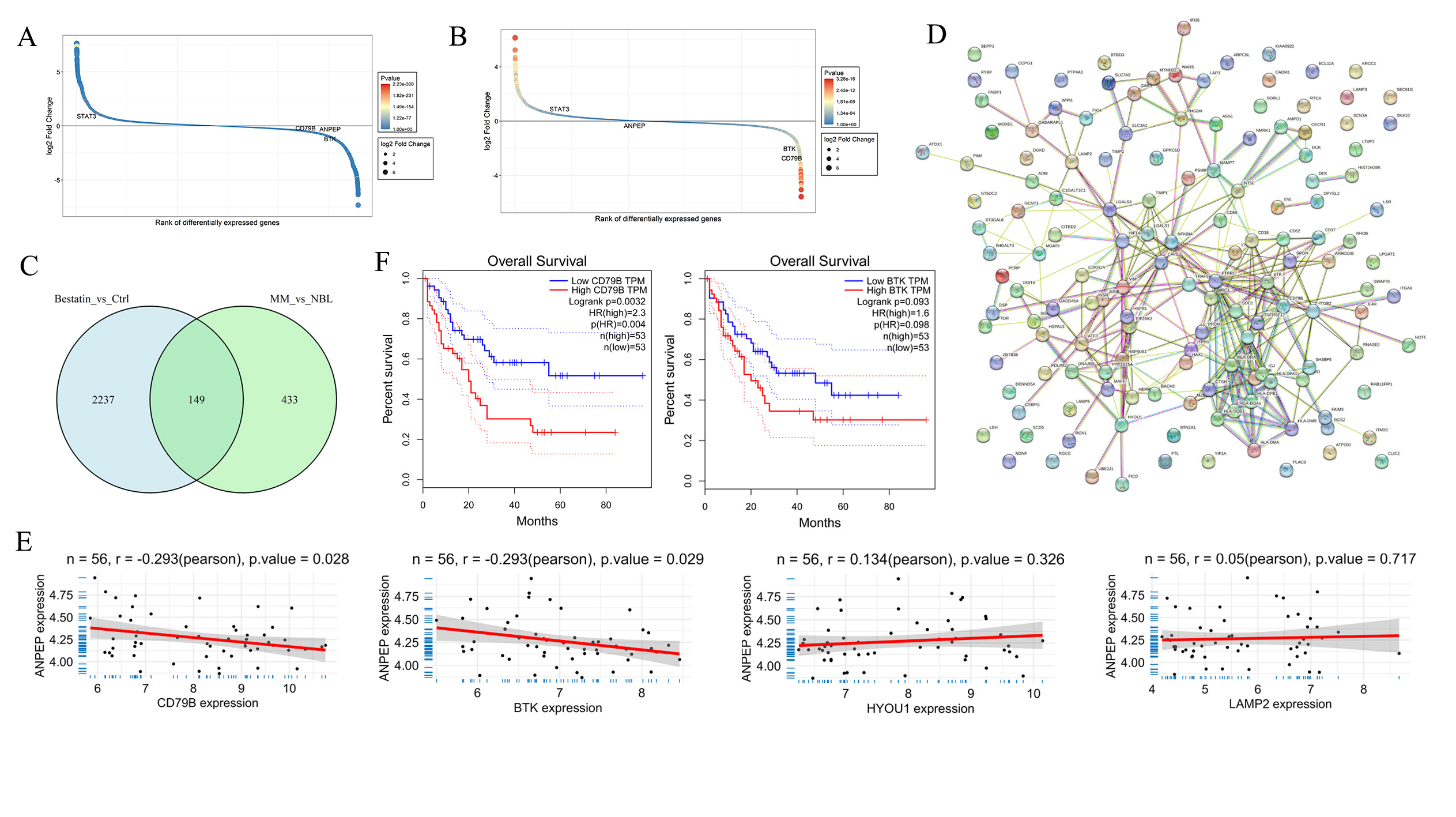

Supplement: Supplementary file 1 [file cells-15-00949-s001.zip › Supplementary Figure S1.jpg]

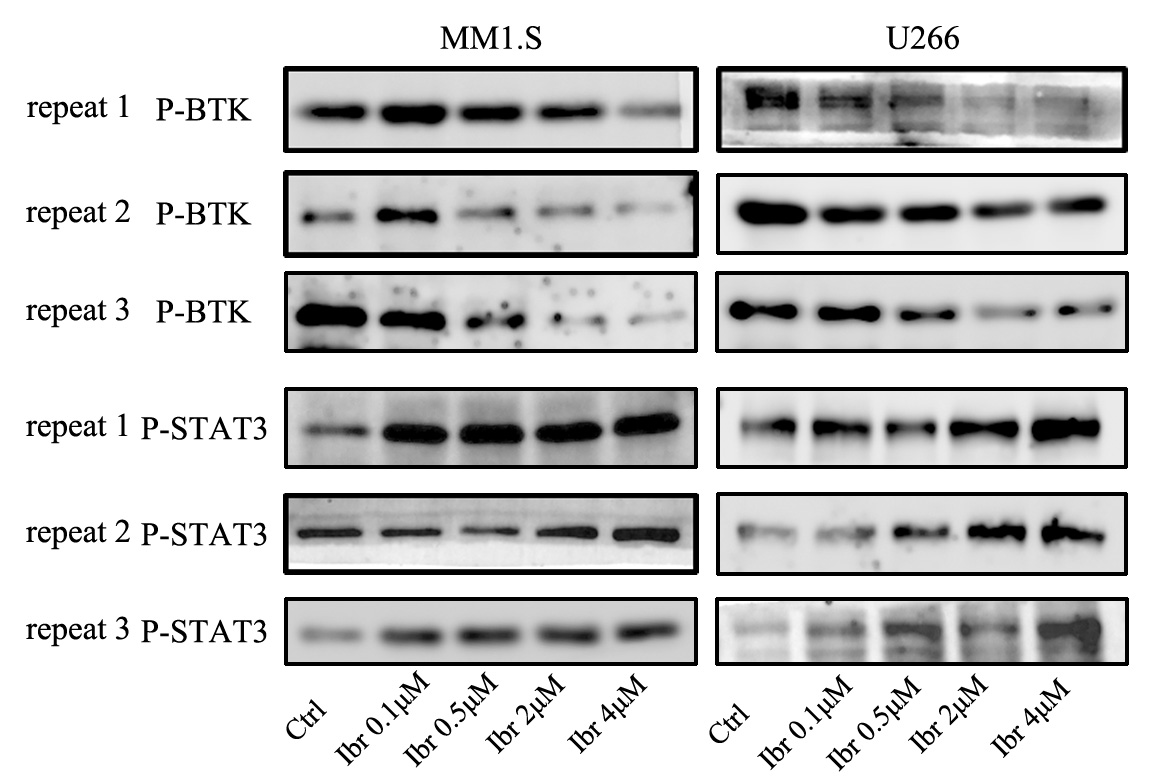

Supplement: Supplementary file 1 [file cells-15-00949-s001.zip › Supplementary Figure S2.jpg]

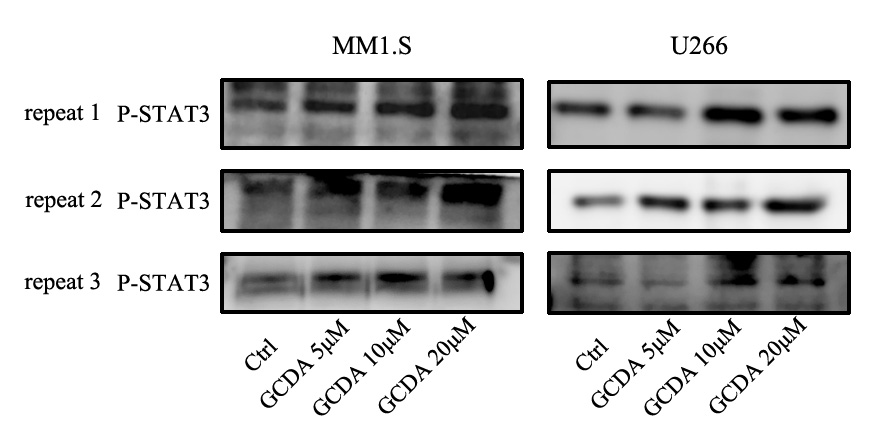

Supplement: Supplementary file 1 [file cells-15-00949-s001.zip › Supplementary Figure S3.jpg]

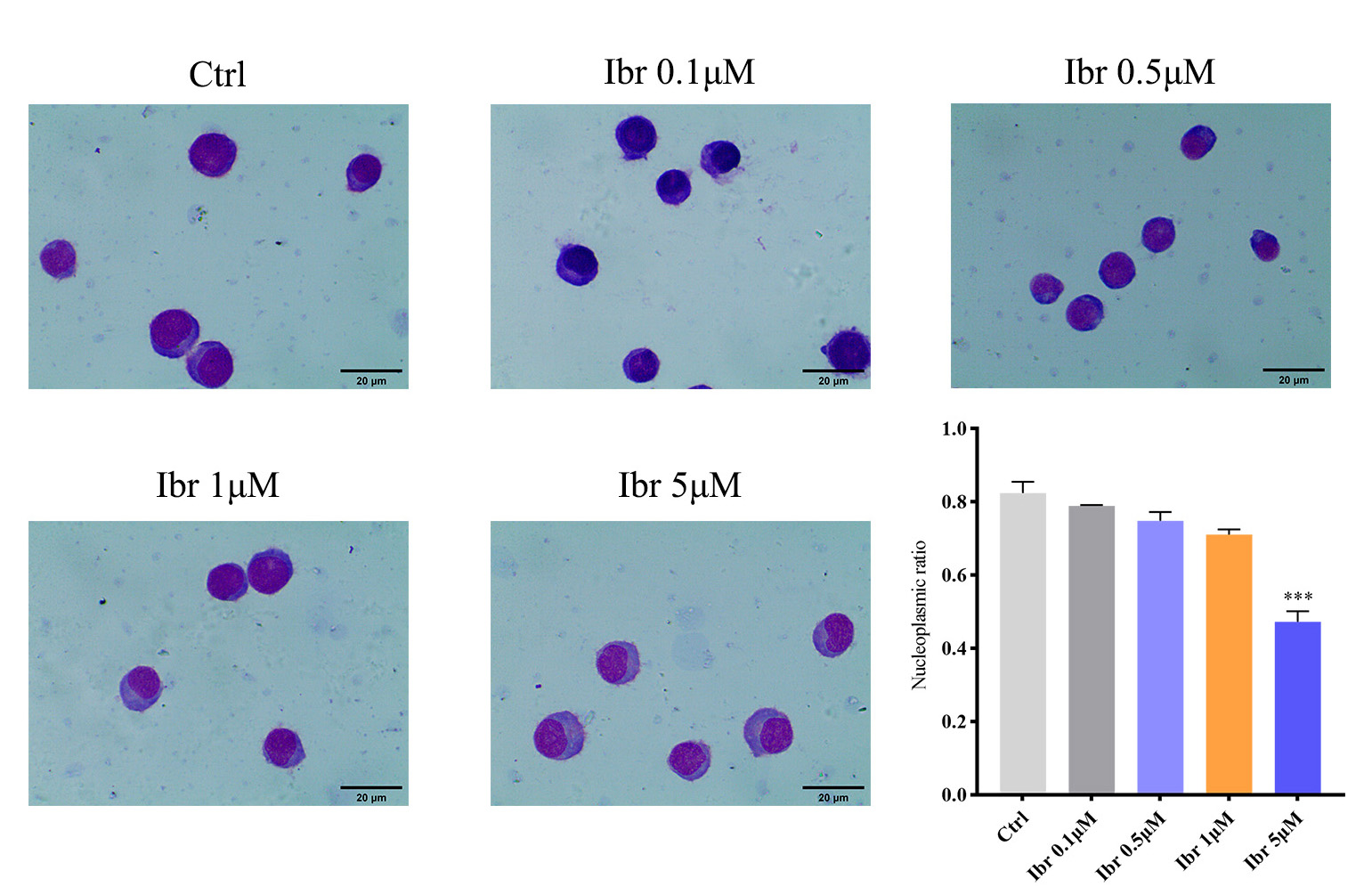

Supplement: Supplementary file 1 [file cells-15-00949-s001.zip › Supplementary Figure S4.jpg]

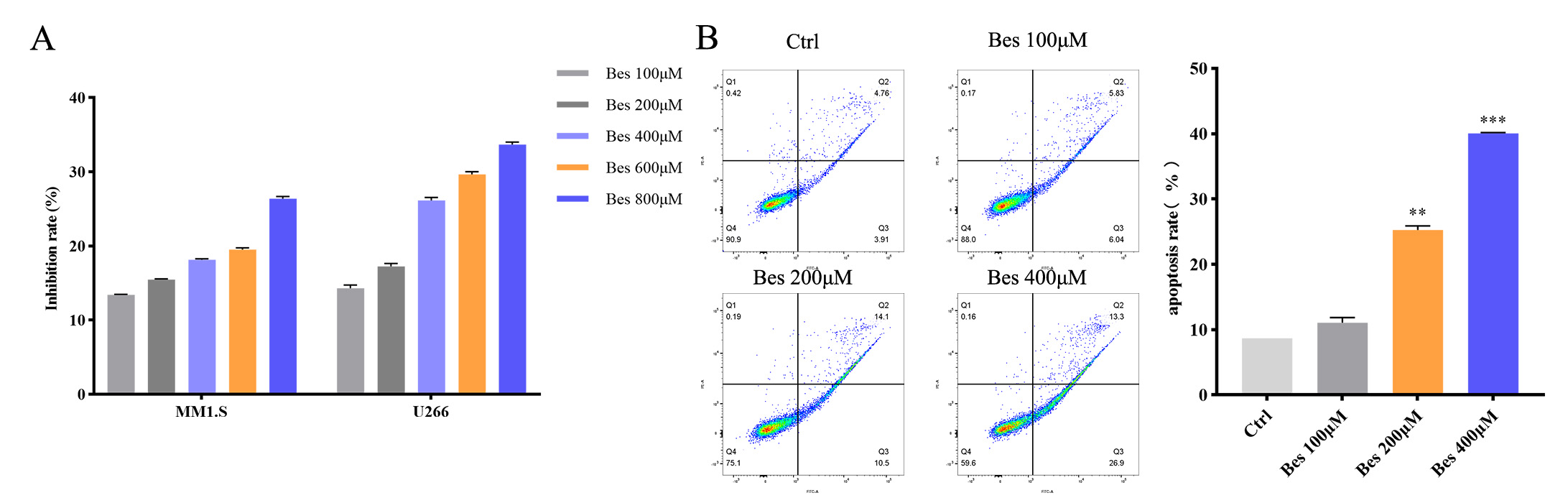

Supplement: Supplementary file 1 [file cells-15-00949-s001.zip › Supplementary Figure S5.jpg]
